# Paraventricular Nucleus Modulates Excitatory Cardiovascular Reflexes during Electroacupuncture

**DOI:** 10.1038/srep25910

**Published:** 2016-05-16

**Authors:** Stephanie C. Tjen-A-Looi, Zhi-Ling Guo, Liang-Wu Fu, John C. Longhurst

**Affiliations:** 1 Susan Samueli Center for Integrative Medicine School of Medicine, Univ. of California, Irvine, CA 92697, USA

## Abstract

The paraventricular nucleus (PVN) regulates sympathetic outflow and blood pressure. Somatic afferent stimulation activates neurons in the hypothalamic PVN. Parvocellular PVN neurons project to sympathoexcitatory cardiovascular regions of the rostral ventrolateral medulla (rVLM). Electroacupuncture (EA) stimulates the median nerve (P5-P6) to modulate sympathoexcitatory responses. We hypothesized that the PVN and its projections to the rVLM participate in the EA-modulation of sympathoexcitatory cardiovascular responses. Cats were anesthetized and ventilated. Heart rate and mean blood pressure were monitored. Application of bradykinin every 10-min on the gallbladder induced consistent pressor reflex responses. Thirty-min of bilateral EA stimulation at acupoints P5-P6 reduced the pressor responses for at least 60-min. Inhibition of the PVN with naloxone reversed the EA-inhibition. Responses of cardiovascular barosensitive rVLM neurons evoked by splanchnic nerve stimulation were reduced by EA and then restored with opioid receptor blockade in the PVN. EA at P5-P6 decreased splanchnic evoked activity of cardiovascular barosensitive PVN neurons that also project directly to the rVLM. PVN neurons labeled with retrograde tracer from rVLM were co-labeled with μ-opioid receptors and juxtaposed to endorphinergic fibers. Thus, the PVN and its projection to rVLM are important in processing acupuncture modulation of elevated blood pressure responses through a PVN opioid mechanism.

Electroacupuncture (EA) stimulation with low current and low frequency (2–4 mA, 2 Hz, 0.5 ms) decreases blood pressure and elevates plasma norepinephrine in patients with mild to moderate hypertension^1^. Experimental studies show that prolonged stimulation of somatic nerves with EA alters activity of the autonomic nervous system. For instance in an animal model with partial coronary artery occlusion, 30 min. of EA stimulation of median nerves located below at P5-P6 acupoints reduces the extent of myocardial ischemia induced by visceral-cardiovascular pressor reflexes and hence the imbalance between oxygen supply and demand^2^,^3^. Our previous studies also have shown that EA modulates these sympathoexcitatory cardiovascular reflex responses, in part, through an opioid mechanism in the rostral ventrolateral medulla (rVLM). We also have demonstrated that the inhibitory action of EA involves central pathways that extend from the hypothalamus to the medulla, including both the arcuate nucleus (ARC) and the rVLM^4^,^5^,^6^,^7^,^8^.

Hypothalamic parvocellular neurons in the paraventricular nucleus (PVN) regulate cardiovascular function and sympathetic outflow^9^,^10^,^11^. The PVN projects directly to sympatho-excitatory cardiovascular regions of the rVLM and to a lesser extent to the intermediolateral column of the spinal cord^12^,^13^,^14^,^15^. There are many sources of afferent input to the PVN that are activated by a number of diverse conditions such as exercise, chronic overcrowding stress, reflex input from the heart during atrial balloon distension and coronary ligation^16^,^17^,^18^,^19^,^20^. Somatic afferent stimulation activates c-Fos expression in the PVN^21^ but not much is known about its physiological actions during the modulatory actions of acupuncture in regulation of cardiovascular neurons. In addition, the PVN is rich in opioids. For instance, enkephalin and β-endorphin are present in the PVN and participate in regulation of cardiovascular function^22^,^23^,^24^. We therefore examined the possibility that stimulation of somatic afferents with acupuncture modulates PVN cardiovascular activity through an opioid mechanism.

We have shown that the rVLM, an important nucleus in regulation of sympathetic outflow, is inhibited by EA. Endorphin, by activating μ-opioid receptors in the rVLM, inhibits sympathetic outflow and decreases sympathetic excitatory response induced by activation of visceral afferents with application of bradykinin (BK) on the gallbladder through activation of the splanchnic nerve^2^,^25^. Cardiovascular premotor sympathoexcitatory rVLM neurons that receive convergent input from afferents innervating the gallbladder, baroreceptors and median nerves have been shown to process EA-cardiovascular inhibition^26^,^27^. These rVLM neurons display prolonged inhibition lasting beyond the 30-min of EA stimulation at P5-P6 (sensitive to EA) and thus contribute to the reduction of sympathoexcitatory visceral cardiovascular responses. We do not know if rVLM cells are influenced by projections from PVN neurons during EA inhibition of pressor responses. We hypothesized in the present study that EA modulates autonomic cardiovascular responses, in part, through an opioid mechanism in the PVN. We also examined the possibility that EA inhibits the pressor responses and activity in rVLM, in part, through a PVN-rVLM projection. These data have been published in preliminary form^28^.

## Results

### Physiological studies in cats

#### Effects of naloxone in the PVN on EA modulation of pressor reflexes

**Figure 1:**
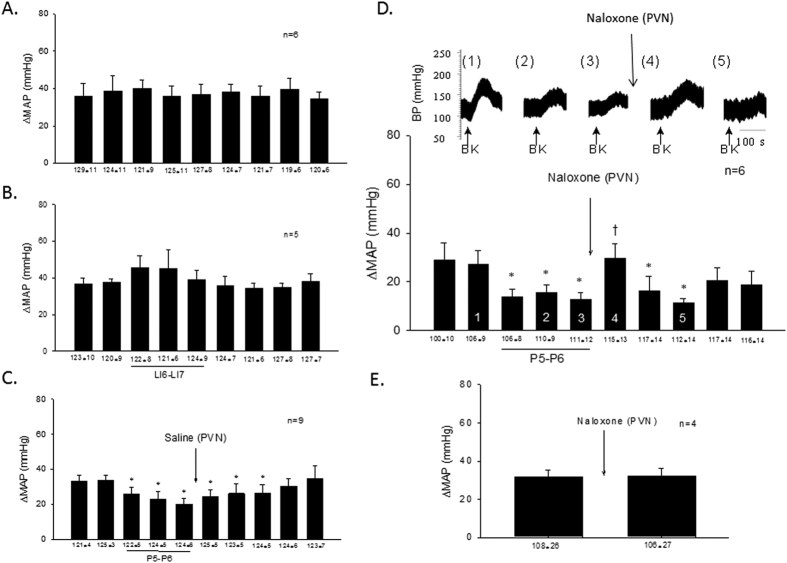
Excitatory cardiovascular reflex responses inhibited by EA through opioids in PVN. Repeated gallbladder stimulation with bradykinin every 10-min evoked consistent mean arterial pressure (MAP) responses (Panel (**A**)). Electroacupuncture (EA) at control acupoints LI6-LI7 did not the inhibit sympathoexcitatory reflex responses (Panel (**B**)) while 30-min stimulation at P5-P6 reduced these gallbladder-induced reflex increases in blood pressure (Panel (**C**)). The effect of acupuncture lasted for a prolonged period of time during and after 30-min EA application. Saline microinjection into the PVN did not alter EA’s action (Panel (**C**)) while naloxone reversed the inhibitory action of acupuncture (Panel (**D**)). Numbers shown in bars of histogram (Panel (**D**)) correspond with blood pressure tracings shown above (Panels D1-5). EA reduced excitatory cardiovascular responses that were reversed by naloxone microinjection into the PVN (Panel D4). In the absence of EA, naloxone did not affect the pressor responses (Panel (**E**)). Mean and SEM below bars display baseline blood pressures. (*), indicates significant reduction in reflex responses compared with changes in MAP before onset of EA. (†), Indicates significant reversal of the EA inhibitory response compared to immediately preceding BK stimulation during EA.

The cardiovascular sympathoexcitatory responses to repeated stimulation of the gallbladder with BK every 10 min were consistent without EA and with EA at control acupoints LI6-LI7 in eleven animals (Fig. 1A,B). Sympathoexcitatory cardiovascular responses were reduced by EA applied at P5-P6 and remained inhibited after microinjection of saline into the PVN in nine subjects (Fig. 1C). On the other hand, blockade of opioid receptors in the PVN reversed the inhibitory effect of EA but did not influence the pressor responses in the absence of EA in ten subjects (Fig. 1D,E). Heart rate and mean arterial pressure (MAP) with and without naloxone before the onset of each reflex response were consistent throughout the protocols.

#### PVN neuronal activity during EA

**Figure 2:**
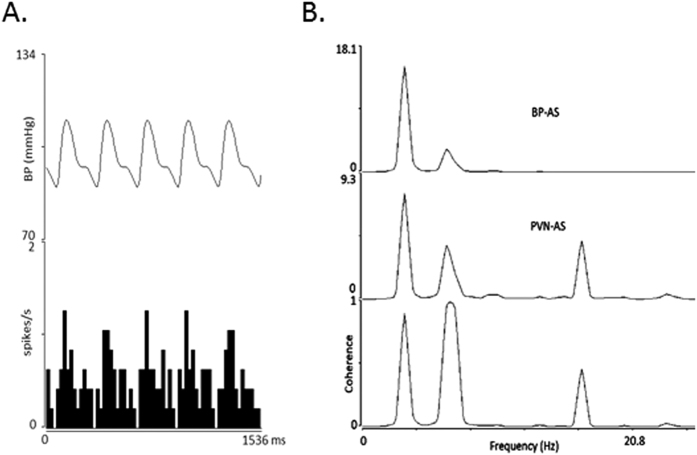
PVN neuron characterized with inputs from splanchnic and median nerves was evaluated for cardiac rhythmicity. Panel A displays strong relationship between PVN discharge and blood pressure (bin = 12 ms) and Panel B shows a strong coherence (between PVN neuronal activity and blood pressure) of 0.89 at a frequency of 3.25 Hz. AS = autospectrum.

**Figure 3:**
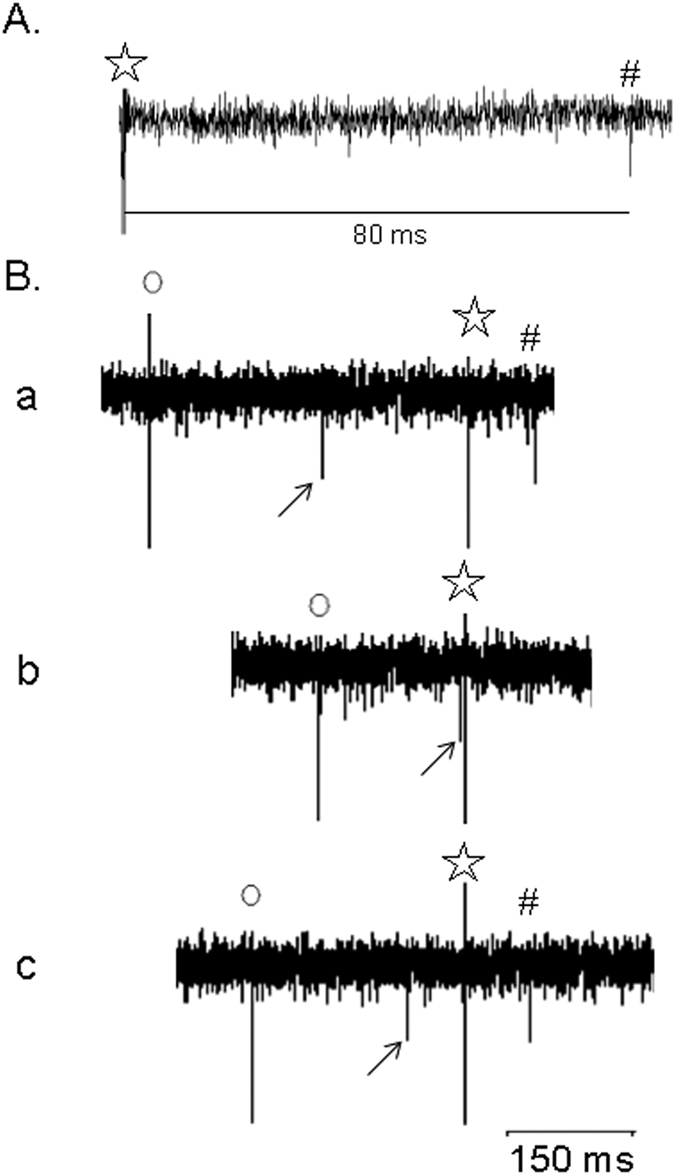
PVN neuron receiving input from splanchnic and median nerves was evaluated for projection to rVLM. Stimulation of the rVLM (⋆) antidromically evoked a PVN spike (#) (Panel (**A**)). Collision testing (conduction velocity = 0.3 m/s, latency = 80 ms and refractory period = 6 ms) is shown in Panels (**A**,**B**). Tracings a and c in Panel (**B**) display from left to right stimulation artifact (o) of median nerve, median nerve-evoked orthodromic spike (arrow), stimulation artifact of rVLM (⋆) followed by the antidromically rVLM evoked PVN spike (#). In tracing b, the median nerve-evoked orthodromic spike (arrow) to the left of the rVLM-artifact (⋆) canceled the antidromically activated PVN spike (note the absence of a spike to the right of the rVLM artifact).

**Figure 4:**
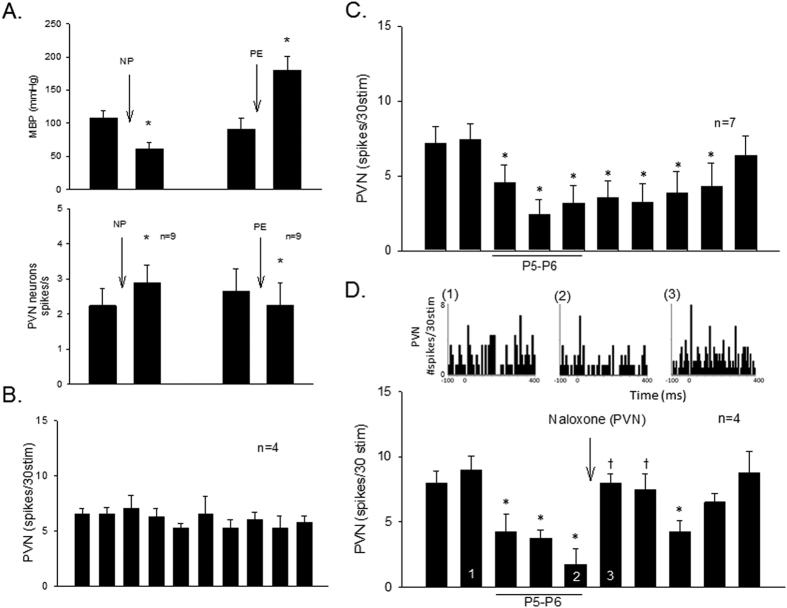
Cardiovascular PVN neurons evaluated with baroreceptor activation and for EA-sensitivity. Decreased mean blood pressure (MBP, Panel (**A**), top) increased PVN activity (Panel (**A**), bottom) while elevated MBP (Panel (**A**), top) reduced number of PVN spikes (Panel (**A**), bottom). Symbol (*) in Panel (**A**), top and bottom histograms, indicates significant changes in MAP and PVN activity after administration of nitroprusside (NP) and phenylephrine (PE). Panel B displays consistent evoked PVN activity with repeated splanchnic nerve stimulation every 10-min. EA for 30-min at P5-P6 reduced the splanchnic evoked PVN responses for at least 70 min, Panel (**C**). Opioid receptor blockade with naloxone reversed EA-inhibition of splanchnic evoked PVN activity (Panel (**D**)). Peristimulus histograms (Panels D1-3) display discharge activity of a cardiovascular PVN neuron before and during 30-min EA at P5-P6 stimulation and following naloxone microinjection that correspond with numbers in bar histogram (Panel (**D**)). Symbol (*) in Panels (**C**,**D**) indicates significant reduction in evoked neuronal responses compared with activity before onset of EA. Symbol (†) in Panel (**D**) indicates significant reversal of PVN evoked activity compared to pre-blockade during the effects of EA.

Seventeen PVN neurons were evaluated to define their characteristics including responses to visceral and somatic afferent stimulation, cardiac rhythmicity, baroreceptor activation, direct projection from the PVN to the rVLM using collision testing, and EA-sensitivity. All PVN cells demonstrated a cardiac rhythmicity (Fig. 2A,B). The average coherence was 0.86 ± 0.03 at a blood pressure pulse frequency of 3.57 ± 0.4 Hz, representing the average heart rate of 214 ± 25 beats/min. Three of the PVN neurons examined for PVN-rVLM projections were evoked by antidromic stimulation (Fig. 3). These three neurons also were EA-sensitive. All cells were responsive to baroreceptor loading or unloading (Fig. 4A). The PVN neurons displayed a baseline activity of 3.0 ± 0.4 spikes/s.

Fourteen of the 17 cardiovascular PVN cells that received input from both the splanchnic and median nerves either could not be antidromically stimulated from the rVLM (n = 10) or were not tested for antidromic evoked activity (n = 4). Four of these 14 cells demonstrated consistent evoked activity with repeated splanchnic nerve stimulation every 10-min (Fig. 4C). Ten other neurons were tested for EA-sensitivity, i.e., response to 30-min somatic nerve stimulation at the P5-P6 acupoints located over the median nerves. Eight cells were EA-sensitive (see Methods) while two neurons were not sensitive to EA. A total of 11 PVN neurons, including the three neurons antidromically activated from the rVLM, were responsive to 30-min EA. Splanchnic nerve evoked activity was reduced for at least 70 min by EA (Fig. 4C,D). Naloxone reversed the EA-modulated activity in four of the 11 PVN cells (Fig. 4D) while the receptor blockade did not decrease (from 9.3 ± 0.88 to 8.3 ± 1.2 spikes/30stim, P = 0.47) the splanchnic evoked PVN activity in the absence of EA. Saline microinjection did not influence the effects of EA. The activity of one individual PVN neuron analyzed using peristimulus histograms was decreased during and at least 30 min after EA application (Fig. 4D, Panels 1 and 2). The reversal of action of EA following naloxone is shown in Fig. 4D, Panel 3.

#### EA inhibition of rVLM activity influenced by opioid receptor blockade in PVN

The rVLM neurons were evaluated for their responses to visceral, somatic and cardiovascular convergence and cardiac rhythmicity. Only cells that received median nerve input and were evoked by stimulation of the splanchnic nerve were evaluated. We observed a strong relationship between neuronal activity and arterial blood pressure in 20 cells. Baseline activity was 3.25 ± 0.9 spikes/s. The average coherence was 0.88 ± 0.03 at a blood pressure pulse frequency of 3.2 ± 0.3 Hz, representing the average heart rate of 192 ± 18 beats/min. We then examined the responses of cells to altered baroreceptor activity input using nitroprusside or phenylephrine. Nitroprusside increased discharge activity from 2.7 ± 0.4 to 6.7 ± 0.8 spikes/s while phenylephrine reduced activity from 3.9 ± 0.9 to 2.1 ± 0.5 spikes/s.

**Figure 5:**
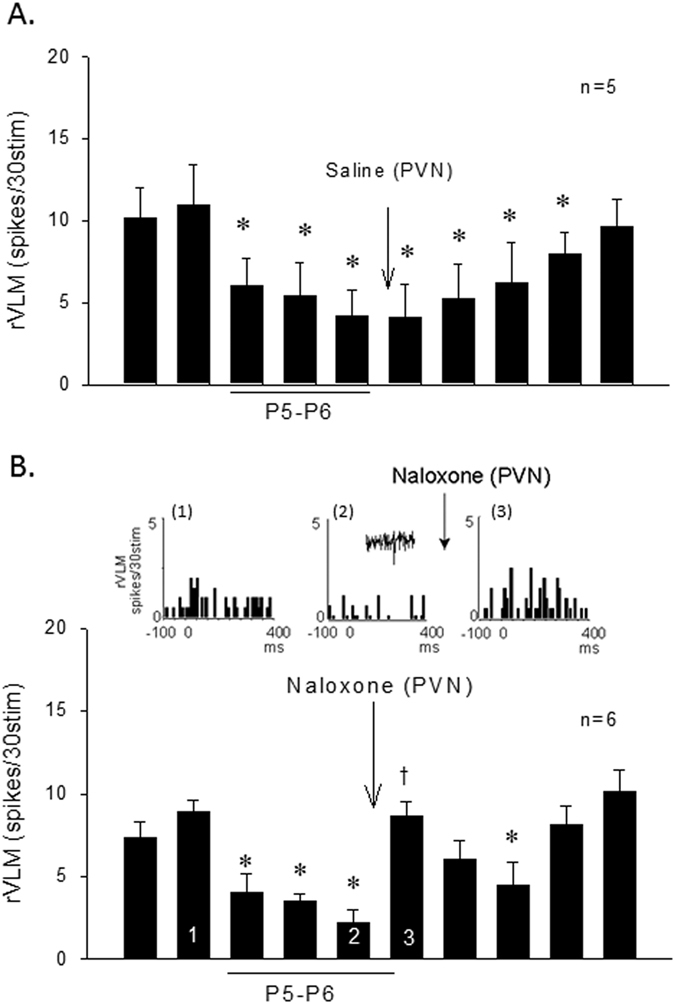
Cardiovascular rVLM neurons receiving baroreceptor, splanchnic and median afferent convergent input responsive to EA inhibition of splanchnic evoked activity through an opioid mechanism. While microinjection of saline did not affect the inhibitory action of EA (Panel (**A**)), the rVLM evoked activity was reduced during and after application of EA at P5-P6 (Panel (**B**)). Bar histogram shows group data of rVLM neurons with reduced evoked activity during EA application, while opioid blockade with naloxone in the PVN reversed the reduced activity (Panel (**B**)). Peristimulus histograms (Panels B1 and 2 shown above bar histogram in Panel (**B**)) display change in splanchnic-evoked activity of an rVLM neuron before and during 30-min EA at P5-P6 stimulation. Panel B3 displays the rVLM activity in response to microinjection of naloxone into the PVN. (*), Indicates significant reduction in evoked neuronal responses compared with activity before onset of EA. (†), Indicates significant reversal of the EA inhibitory response compared to response immediately preceding blockade during EA.

We further examined these 20 rVLM cells to determine the contribution of the PVN-rVLM connection during EA modulation of splanchnic induced reflex responses. We observed consistent evoked rVLM activity (ranges from 10.8 ± 4.0 to 16.7 ± 4.1 spikes/30stim, P = 0.77) with repeated stimulation of splanchnic nerves every 10 min in five cells that were not subjected to EA. The consistent evoked activity of four rVLM cells was not affected (from 7 ± 2.7 to 7 ± 2.1 spikes/30stim, P = 0.45) following microinjection of naloxone into PVN. In five other rVLM neurons, EA at P5-P6 reduced the evoked activity while saline microinjection into the PVN did not alter the inhibitory effect of EA (Fig. 5A). On the other hand, splanchnic nerve evoked discharge of six other rVLM neurons inhibited by EA was increased following microinjection of naloxone into PVN (Fig. 5B). Activity of an individual rVLM neuron analyzed with peristimulus histograms was increased following naloxone delivery into the PVN (Fig. 5B, Panel 3). While the evoked activity was reduced by EA, basal activity was not influenced by acupuncture.

#### Histology

**Figure 6:**
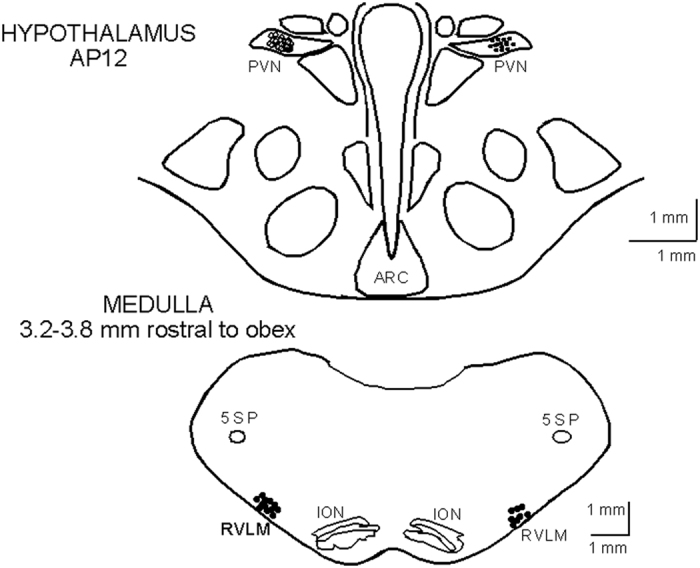
Composite map displays sites of microinjection, recording and iontophoresis in the PVN and rVLM in cat. For ease of presentation, extracellular recording and iontophoresis sites in the PVN (coronal section AP12) are displayed on the right (•) and microinjections sites (o, ⊗) on the left. Microinjections into PVN to examine projection to rVLM are shown with ⊗. Recording sites in rVLM rostral to obex are shown on both right and left. RFN, retrofacial nucleus; 5SP, alaminar spinal trigeminal nucleus; ION, inferior olivary nucleus; ARC, arcuate nucleus.

Sites of microinjection and recording marked with Chicago blue dye were confirmed histologically. Sites were determined by the location of microinjection tracks and dye spots. PVN sites were confirmed to be 1.2–1.67 mm lateral to the midline and 5.2–6.0 mm from the ventral surface. In the rVLM (lateral to the inferior olivary nucleus and ventral and medial to the facial and retrofacial nuclei), the sites were located 3.0–3.9 mm lateral from the midline and 0.1–0.9 mm from the ventral lateral surface. Figure 6 represents a composite map of all sites closely matched with the cat brain atlases^29^,^30^.

### Anatomical studies in rats

#### Retrograde labeling and opioid immunohistochemical staining

Two animals were eliminated from this study since the sites for microinjection were found to be outside the rVLM. Thus three rats in which the unilateral microinjection site of the retrograde tracer was found inside the rVLM, as described previously^5^,^8^, were included in this study. The locations of injected tracer in the medulla closely matched the coordinates of the rVLM as defined by Paxinos and Watson's atlas for the rat^31^. The microinjection sites in the rat were located 2.0–2.6 mm lateral from the midline and 0.2–0.6 mm from ventral surface of the medulla. They were lateral to the paragigantocellularis nucleus, ventral to the Botzinger complex, and medial to the VII cranial nucleus at the high rostral level^5^,^31^. The areas of distribution of the tracer in the rVLM in dorso-ventral planes ranged approximately 0.14 mm × 0.20 mm and at rostral-caudal extension from 0.43–0.48 mm.

We observed that neurons labeled with the retrograde microsphere tracer from the rVLM consistently were distributed rostrally and caudally throughout the PVN of the three rats. The labeled neurons were located in medial, lateral and posterior subdivisions of PVN in the rat, mainly at levels from Bregma −1.72 to −2.04 mm^31^. Approximately two-third the neurons labeled with microspheres in the PVN were found to be located ipsilateral to the injection site in the rVLM.

**Figure 7:**
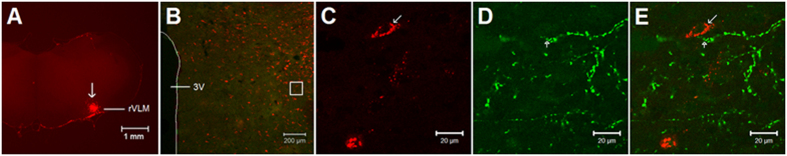
Panel (**A**): fluorescent image demonstrating the microinjection site of the retrograde microsphere tracer in the rVLM (Bregma −12.48 mm, Paxinos and Watson’s rat brain atlas). Arrow indicates the injection site. Scale bar represents 1 mm. Panels (**B**–**E**): confocal microscopic images demonstrating the labeling for a retrograde microsphere tracer injected into the rVLM (Panels **C**) and endorphinergic neuronal processes (Panel (**D**)) in paraventricular nucleus (PVN; Bregma −1.92 mm, Paxinos and Watson’s rat brain atlas) of rats. Panel E is the merged image from Panels (**C**,**D**). Panel (**B**): low-power photomicrograph showing third ventricle (3 V) and distribution of the retrograde tracer and endorphinergic neuronal processes in the PVN (red and green). Magnified region marked with the box in Panel B is shown in Panels (**C**–**E**). Arrows in Panels C and E indicate a neuron labeled with the retrograde tracer originating from the rVLM. Arrow heads in (**D**,**E**) indicates endorphinergic neuronal processes (green). In Panel (**E**), a PVN cell labeled with retrograde tracer indicated by an arrow (red) is in close proximity to fibers labeled with β-endorphins indicated by an arrowhead (green). Scale bars in A, B and C-E represent 1 mm, 200 μm and 20 μm, respectively.

**Figure 8:**
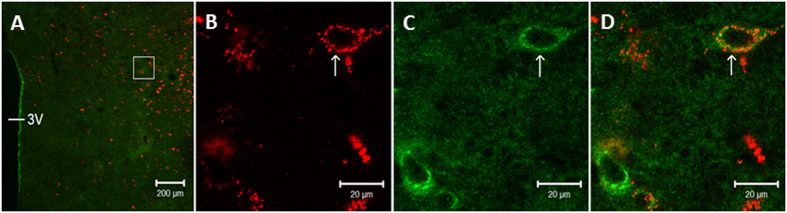
Confocal microscopic images demonstrating double-labeling with a retrograde microsphere tracer injected into the rVLM and μ-opioid receptors in the PVN of rats (Panels (**A**,**D**)). Panel A: low-power photomicrograph; Panels (**B**–**D**): magnified region shown within box in (**A**). Panel (**D**) is merged image from Panels (**B**,**C**). Arrows in (**B**,**C**) indicate a neuron labeled with the retrograde tracer (red) originating from the rVLM and μ-opioid receptors (green). An arrow in Panel (**D**) indicates the neuron double-labeled with the tracer and μ-opioid receptors (yellow and orange). 3 V: the third ventricle. Scale bars in A and B–D represent 200 and 20 μm, respectively.

In all three rats, β-endorphin fibers and associated μ-opioid receptors were distributed bilaterally throughout the caudal and rostral PVN. Cell bodies stained with tracer were positioned closely (the distance between the two labels was less than 0.5 μm) to β-endorphin neuronal processes, as demonstrated in Fig. 7. They also were co-labeled with μ-opioid receptors as shown in Fig. 8. The average number of rVLM projecting PVN neurons was estimated to be about 165 per section. Neural processes labeled with β-endorphin were in close apposition to the majority of rVLM projecting PVN neurons labeled with the retrograde tracer. Approximately 30% of the retrograde tracer-labeled PVN neurons were co-localized with μ-opioid receptors.

## Discussion

We have demonstrated that the long-lasting inhibitory effect of point specific EA on reflex induced elevated blood pressure is related to the modulation of cardiovascular neurons in the rVLM^27^,^32^. As demonstrated previously^5^,^7^,^8^, this inhibition lasts for over an hour after termination of EA in anesthetized animals. The prolonged influence of EA modulation of rVLM activity involves a long-loop supraspinal pathway that includes the ARC, ventrolateral periaqueductal gray (vlPAG), nucleus raphé pallidus (NRP) and rVLM^5^. We currently show that EA reduces activity in the rVLM through an opioid mechanism in the PVN that is rich in endorphinergic fibers and μ-opioid receptors. Collectively, these findings suggest that the PVN serves as another important hypothalamic nucleus contributing to the prolonged inhibition of cardiovascular reflex responses by EA.

The neurons in the PVN and rVLM are known to be important in the regulation of cardiovascular function^9^,^33^ and, as demonstrated in present study, participate in the EA modulation of visceral sympathoexcitatory cardiovascular reflex responses. The PVN is activated in a number of diverse cardiovascular related conditions including exercise, chronic overcrowding stress, cardiovascular deconditioning, reflex input from the heart during atrial balloon distension and coronary ligation^16^,^17^,^18^,^19^,^20^,^34^. Neurons within the parvocellular region of the PVN influence baroreceptor blood pressure regulation^35^,^36^,^37^,^38^,^39^ and sympathetic activity^10^. PVN neurons project to the intermediolateral column (IML) of the spinal cord, rVLM and the nucleus tractus solitarius^40^,^41^,^42^. Shafton *et al*. reported that the number of PVN-rVLM neurons is an average of sevenfold greater than the number of PVN-IML neurons^43^ providing a rationale to focus on PVN-rVLM projections in regulation of blood pressure responses during acupuncture. However, we cannot exclude a role for PVN connections through other nuclei during EA cardiovascular modulation. The present study shows that splanchnic nerve activated PVN and PVN-rVLM neurons are barosensitive and show strong correlation with the cardiac cycle, consistent with the observations made by Chen *et al*.^15^. A new observation is that splanchnic-evoked PVN activity is inhibited during and following 30 min of somatic afferent stimulation with EA. Furthermore, the present study demonstrates for the first time that PVN neurons projecting to rVLM are modulated during EA-inhibition of cardiovascular reflex sympathoexcitatory responses for a prolonged period of time.

We used rats rather than cats in the present anatomical studies to confirm the presence of rVLM projecting PVN neurons. Data obtained in rats most likely also apply to cats. Our laboratory has shown that, in addition to the similar cardiovascular physiological action of EA in the two species^44^, the anatomic circuitry in the cardiovascular responses in rats and cats are virtually identical^5^,^8^,^45^,^46^. Using retrograde tracing and immunohistochemistry the current study shows the potential for a relationship between PVN neurons and opioids in the parvocellular region of the PVN. In this regard, we detected endorphinergic fibers positioned close to these rVLM-projecting PVN neurons (Fig. 7). We also have found μ-opioid receptors in PVN neurons that project to the rVLM (Fig. 8). As the PVN is rich in opioidergic neurons, endorphin and enkephalin fibers, and receptors^22^,^23^,^24^,^47^, we examined the role of opioids in PVN during central processing of the actions of EA.

Our present data shows that blockade of opioid receptors in PVN reverses EA inhibition of both evoked PVN and rVLM neuronal responses and elevated blood pressure. Opioid receptor blockade in PVN does not affect the cellular evoked activity in the PVN and rVLM in the absence of EA. In rat hypothalamic brain slice preparation, opioids reduce neuronal activity of PVN neurons^48^,^49^ supporting the concept that EA somatic stimulation through opioid mechanisms reduces elevated PVN neuronal activity. Sapru *et al*. show that neurochemicals like β-endorphin in the PVN influences rVLM activity^47^,^50^ further supporting our current findings on actions of EA through a PVN and rVLM connection. Thus present study has shown that the opioids in the PVN contribute to the inhibition of cardiovascular PVN cells that, in turn, reduces rVLM activity and hence cardiovascular reflex responses during the effects of EA.

Different regions in the hypothalamus regulate cardiovascular function including the ARC and the PVN^15^,^50^,^51^,^52^. The present findings further clarify the interaction of somatic nerves during EA with the cardiovascular regions in the hypothalamus. We have shown that the ARC is activated during and after 30 min of median nerves EA application^5^,^51^ to decrease elevated blood pressure reflex responses. Endorphinergic cell bodies co-labeled with EA-activated c-Fos expression are observed in the ARC^5^. The ARC is rich in β-endorphinergic neurons that project to the PVN^47^,^50^. Activation of the ARC also activates PVN opioid receptors^47^,^50^. The present new findings show that the PVN is inhibited during and after EA stimulation through an opioid mechanism. These observations suggest that EA-evoked increased activation of ARC cells expressing endorphin may be involved in the PVN during central processing of the actions of EA. Future investigation is required to determine if ARC targets the PVN to modulate pressor responses during acupuncture.

In summary, our results suggest that the long-lasting effect of EA at P5-P6 on visceral reflex increases in blood pressure is related, in part, to the inhibition of neurons in the hypothalamus PVN through a PVN-rVLM projection. EA inhibition involving the PVN modulates cardiovascular rVLM activity and, in turn, blood pressure responses through an endorphinergic opioid mechanism in the PVN.

## Materials and Methods

### Surgical Procedures

The animal use and care committee at the University of California, Irvine, approved all surgical and experimental protocols used in this study. All procedures were carried out in accordance with the US Society for Neuroscience and the National Institutes of Health guidelines. The minimal possible number of cats was used to obtain reproducible and statistically significant results. Cats of both sexes (3–4 kg) were anesthetized initially with an injection of ketamine (40 mg/kg, im). A femoral vein and artery were cannulated respectively for administration of drugs and fluids and measurement of arterial blood pressure (Statham P 23 ID, Oxnard, CA, USA). An intravenous injection of α-chloralose (50 mg/kg, iv) was administered. Supplemental α-chloralose (5–10 mg/kg, i.v.) was given if the animals exhibited a corneal reflex, withdrew a limb in response to a noxious stimulus or displayed an unstable respiratory pattern or blood pressure. Heart rate (HR) was derived from the arterial pressure pulse by a biotech (Gould Instruments, Cleveland, OH, USA). Intubation of the trachea enabled artificial respiration (Harvard pump, model 662, Ealing, South Natick, MA, USA). Arterial blood gases were measured (Radiometer, Model ABL-5, Westlake, OH, USA) and maintained within the normal physiological range (PO_2_, 100–150 mmHg; PCO_2_, 28–35 mmHg; pH 7.35–7.45) by intravenous administration of 8% sodium bicarbonate or by adjusting the ventilator. Body temperature was kept between 36 and 38 °C, using a heating pad and an external heat lamp, as needed.

A lateral laparotomy on the right was performed to expose the surface of the gallbladder or to isolate the splanchnic nerve. This allowed direct placement of a BK presoaked pledget on the serosal surface of the gallbladder^53^. To quantify neuronal activity in the rVLM, we placed a bipolar flexible platinum stimulating electrode around the splanchnic nerve for stimulation of the cardiovascular sympathetic afferent reflex pathway and to evoke rVLM neuronal activity. The stimulating electrode was connected to an isolation unit and a stimulator (Grass, model S88) and was held in place with polyvinyldimethylsiloxone dental impression material (Pentron, Wallington, CT). The abdominal wall was closed to prevent desiccation and heat loss and was reopened only for BK application to the gallbladder. A craniotomy was performed after the animal was stabilized with a Kopf stereotaxic head frame to access the PVN and rVLM.

Microinjection probes consisting of a guide tube with an outer diameter of 0.75 mm and an injection cannula with an inner diameter of 0.4 mm were inserted into the PVN to examine the cardiovascular responses and rVLM neuronal activity. A single barrel glass pipette electrode was used to evaluate PVN or rVLM neuronal activity. The glass pipette was filled with 0.5 M sodium acetate containing 2% Chicago sky blue for later marking (Sigma Chemical, St Louis, MO) and recording electrode. A four-barrel glass pipette electrode was used to iontophorese saline or naloxone and record PVN cellular activity. The barrels of the pipette were filled with a platinum recording wire and 0.5 M sodium acetate containing 2% Chicago sky blue, saline, naloxone, and 4M NaCl to balance the current. Using visual approximation, the microinjection and recording electrodes were positioned perpendicularly to the dorsal surface of the cortex, 9.5 to 11.5 mm rostral to the tentorium, 1.5 mm lateral to the midline and advanced ventrally 21 mm to reach the PVN. We positioned the rVLM recording electrode perpendicularly to the dorsal surface of the medulla, 3–3.5 mm laterally and 3–3.7 mm rostrally relative to the obex and advanced 5 mm ventrally. At the end of the experiment, the recording and microinjection sites were marked with Chicago blue dye for later histological confirmation following recording and administration of drugs into the PVN and rVLM.

### Methods of Blockade

The role of opioids in PVN during effects of EA was evaluated by microinjection and iontophoresis of naloxone (10–100 nM, 50–75 nl, 2 min at 120 nA, Sigma Aldrich) either right or left into the PVN^3^,^46^,^54^,^55^ 10 min after terminating acupuncture stimulation. The timing of injection allowed evaluation of acupuncture’s long-lasting action. Since both 100 and 10 nM naloxone blocked the response to EA, 10 nM was used in 23 of the 24 naloxone blockades in the absence and presence of EA. Microinjection of 75 nl and iontophoresis of saline for 2 min served as the control.

### Stimulating Methods

Repeated stimulation every 10 min of the gallbladder with BK (10 μg/ml) or right splanchnic nerve (2 Hz, 0.4–0.6 mA, 0.5 ms) respectively induced consistent increases in blood pressure or neuronal rVLM activity^27^ and PVN evoked discharge. Gallamine triethiodide (4 mg/kg) was administered intravenously before application of EA or recording neuronal activity to avoid muscle movement during stimulation of somatic nerves. Acupuncture needles were inserted to a depth of about 4 mm, bilaterally, at acupoints P5-P6 to evaluate median nerve input in PVN and rVLM cells as well as EA-sensitivity^2^,^56^. Needles at these acupoints were located 2–3 cm proximal to the flexor crease on the wrist and were separated by 5–7 mm. Bilateral stimulation at acupoints LI6-LI7 activating the superficial radial nerves was used for control. The needles were inserted to a depth of about 1 mm located on the radial side of dorsal surface on the lower one–third of the forelimb. The needles were connected to an isolation unit and stimulator (Grass, model S88) to deliver bipolar stimuli. The stimulation parameters were 2–4 Hz, 2–4 mA using a 0.5 ms pulse^7^. Previous study has shown that these stimulation parameters applied for 30-min at P5-P6 activate Groups III and IV afferents in the median nerve to decrease sympathoexcitatory cardiovascular responses^2^,^56^ and neuronal activity in the medulla^8^,^32^. We applied 30-min of EA to simulate clinical use of this procedure. Separately, a stimulating electrode was inserted into the rVLM to examine using collision testing for rVLM projecting PVN neurons. The rVLM (3 ± 0.5 mm rostral to obex, 3.2 ± 0.3 mm lateral, 6 ± 1 mm deep) was stimulated electrically with 0.2–0.4 mA, 1 Hz and 0.5 ms to antidromically stimulate and evaluate for collision of antidromic and orthodromic-evoked spikes^26^,^27^. The location of the rVLM was determined preliminarily during the experiment with electrical stimulation to evoke small reproducible excitatory responses of 6–11 mmHg. The site of rVLM stimulation was confirmed histologically.

### Extracellular recordings

Single-unit activity of PVN or rVLM neurons was recorded with a platinum electrode inserted in a one-barrel pipette (filled with a solution of 0.5 M sodium acetate containing 2% Chicago sky blue, Sigma Chemical) positioned in the PVN or rVLM. Action potentials were amplified with a preamplifier (Neuroprobe Amplifier Model 1600, A-M Systems, Inc.) attached to a Nerve Traffic Analysis System 662C-3 (Bioengineering, College of Medicine, University of Iowa), filtered (0.3–10 KHz) and monitored with an oscilloscope (Tektronix 2201). Action potentials, blood pressures and heart rates were digitized and analyzed online with a computer and a data acquisition system (SHMU; Shanghai Medical College of Fudan University, China). Conduction velocities of PVN neurons were measured by stimulating the rVLM, recording in the PVN, and estimating the distance between the two ipsilateral sites. To assess the evoked responses to stimulation of splanchnic and median nerves, peristimulus time histograms were constructed for each neuron. Action potentials were analyzed both visually and with the data acquisition program for similar wave shapes, heights, and latency from the time of stimulation. The relationship between PVN or rVLM neuronal activity and blood pressure was assessed by both time and frequency domain analyses using arterial pulse-triggered averaging and coherence analysis^5^,^27^. Examination of baroreceptor afferent input with either nitroprusside (2.5 mg/ml) or phenylephrine (2 mg/ml) provided additional characterization of PVN and rVLM neurons.

### Physiological Experimental Protocols

#### Effects of naloxone in the PVN on EA modulation of pressor reflexes

Increases in blood pressure induced by stimulation of chemosensitive afferents of the gallbladder with one cm^2^ pledgets of filter paper soaked with a solution of BK (10 μg/ml) were repeated nine to ten times to examine influence of EA. After the maximum pressor reflex was achieved, the filter paper was removed and the gallbladder was rinsed three to four times with normal saline to remove BK. The pressor response was calculated as the difference in prestimulus MAP and pressure at the peak of the reflex response. To prevent tachyphylaxis, recovery periods of at least 10 min were provided between applications^8^,^27^. Following two consistent increases in blood pressure responses to stimulation with BK, we began bilateral EA at LI6-LI7 or P5-P6 acupoints using acupuncture needles respectively inserted over the superficial radial or median nerves, five min before the third application of BK; EA stimulation then was continued for 30 min. During EA, BK was applied three times at 10 min intervals. After completion of EA, BK was applied to the gallbladder every 10 min for the next 40 to 50 min. Thus, BK was applied during control (two responses), EA (three responses) and recovery following EA (four to five responses) for a total of ten applications. The role of opioids in the PVN during acupuncture was evaluated by microinjecting naloxone or saline into the PVN 5 min following termination of acupuncture. Previous studies have shown that acupuncture has a prolonged response, typically lasting 60–90 min following its application^5^,^7^,^27^. In the absence of EA stimulation, we tested the repeatability of nine sequential pressor responses induced by BK applied to the gallbladder every 10 min in another group of control animals. The effect of naloxone injected into the PVN on the pressor response also was examined in the absence of EA.

#### PVN neuronal activity during EA

We identified neurons in the PVN using the following criteria. First, PVN neurons were examined for convergence during stimulation of the splanchnic nerve and median nerve at acupoints P5-P6 with peristimulus histograms^46^. Stimuli were applied at a frequency of 2 Hz. We evaluated the evoked neuronal activity over a 15-s period to construct peristimulus time histograms with 30-s stimulation^26^,^46^. Then, spontaneous activity was recorded for 5 min to determine the presence of cardiac rhythmicity using time and frequency domain analyses incorporating arterial pulse-triggered averaging and coherence^8^. To further characterize the cardiovascular responsiveness of these PVN neurons, their response to altered baroreceptor input was evaluated following administration of nitroprusside or phenylephrine. Afterward, we examined the neurons that could be antidromically driven from the rVLM. Using this method, the rVLM was stimulated continuously at 2 Hz, while the recording electrode was lowered slowly at increments of 1 μm through the PVN. Neurons in the PVN that responded to ipsilateral stimulation of the rVLM were evaluated for criteria that indicated antidromic activation. We looked for evidence of a constant latency, stable threshold of the evoked all-or-none response and a faithful response to high rates of stimulation (>200 Hz). Then they were evaluated for the presence of collision of triggered antidromic spikes from the rVLM with either spontaneous or stimulus-induced orthodromic action potentials evoked by stimulating the splanchnic or median nerves. The antidromically driven neurons were examined for faithful responses when the time interval between the stimulus and the evoked action potential was greater than the sum of the latency and the refractory period. Collision was observed when the sum of the latency and the refractory period was greater than the time interval^8^. Conduction velocities of neurons in the PVN that projected to the rVLM were determined by measuring the distance between the site of stimulation in the rVLM and the recording site in the PVN and dividing this number by the latency of conduction of the antidromic response from the rVLM to the PVN. Finally, to examine for the role of opioids and effect of EA on PVN cells, neurons were evaluated for consistent splanchnic nerve electrically evoked responses with and without iontophoresis of naloxone into PVN and prolonged inhibition of the evoked responses with EA (EA-sensitive) applied at P5-P6 acupoints with iontophoresis of naloxone or saline into PVN. The time interval between studies was at least 45 min. Thus, in this latter case, we evaluated PVN neurons for splanchnic and median nerves convergent input during a 30-s period of stimulation and the prolonged EA-inhibition of the neuronal activity during and following the 30-min period of stimulation with EA.

### Influence of blockade of opioid receptors in PVN on EA inhibition of rVLM activity

We looked for cardiac rhythmicity, baroreceptor input, convergence of splanchnic and median nerves and responsiveness to EA in the rVLM. To determine convergence, stimulation of splanchnic or median nerves was applied at 2 Hz. We measured rVLM evoked neuronal activity over a 15-s period with peristimulus histograms^46^. The spontaneous rVLM neuronal firing pattern was evaluated for cardiac rhythmicity using arterial pulse-triggered averaging and coherence analyses^6^,^8^,^57^. To characterize the cardiovascular responsiveness of rVLM neurons, the response to altered baroreceptor input following administration of nitroprusside or phenylephrine was evaluated. These cardiovascular neurons then were studied regarding their long-term inhibitory responses to EA. In this regard, stimulation at P5-P6 for 30-min was used to examine for evidence of prolonged reduction in splanchnic evoked neuronal response to EA. To determine role of opioids in the PVN during EA prolonged inhibition of rVLM neurons, naloxone or saline as a control was microinjected unilaterally into the PVN 5-min after the end of acupuncture application or in the absence of EA.

### Histology

At the end of each experiment, animals were euthanized under deep anesthesia with alpha-chloralose and saturated KCl. The brain and brain stem were removed and fixed in 10% formalin for at least 72 hours. Frozen serial sections (60 μm) of the brain were cut with a freezing microtome (Leica CM 1850). Slices were examined with a microscope (Nikon eclipse 6400) to identify recording and stimulation sites. These sites were reconstructed with Corel Presentation and plotted on coronal sections corresponding to the rostral-caudal planes according to cat brain atlases of the brainstem and hypothalamus^29^,^30^.

### Data analysis

Data are presented as means ± SE. The assumption of normal data distribution was analyzed by the Kolmogorov-Smirnov test. Blood pressure responses to BK were analyzed with a one-way repeated measures analysis of variance, followed post-hoc with the Student-Newman-Keuls test. These tests represented a pair wise multiple comparison procedure. Two-way repeated measures analysis of variance was used for group comparison. Paired-t-test was used to evaluate responses to baroreceptor loading and unloading. We utilized SigmaStat and SigmaPlot software (Jandel Scientific, San Rafael, CA) for statistical analysis and graphing. The level of statistical significance was chosen as P < 0.05.

The efficacy of EA responses was examined by comparing the change of increase of MAP between the second and the following seven to eight applications of BK. We defined the duration of the EA-induced inhibition of the visceral reflex pressor response as the time from the initial decrease after onset of EA to the time when the EA-related decrease in each individual animal was equivalent to or greater than the smallest, but statistically significant, decrease for the overall group. Thus, if the smallest EA-induced decrease in the reflex response for a group was 10 mmHg, then EA-related changes for each animal had to be equivalent to or greater than the number to be considered significant. This procedure allowed us to assess each animal independently rather than simply using the group means to determine the duration of effect. Additionally, group comparisons were made. The responses to repeated application of BK in the absence of EA were compared with blood pressure changes in response to EA at LI6-LI7 or P5-P6. The extent of prolonged inhibition of PVN and rVLM neurons was analyzed by a one-way repeated measures analysis of variance and post-hoc with the Student-Newman-Keuls test to evaluate the decrease in neuronal activity in response to EA at P5-P6. The decrease in evoked neuronal activity during and after acupuncture was relative to the splanchnic evoked activity prior to EA.

Coherence between PVN or rVLM activities and arterial blood pressure was determined with a Fast Fourier Transform (FFT) algorithm^8^. Original data were recorded using a sampling rate of 10,000 Hz; reconstructed data utilized the mean of ten samples (data points) and included assessment of the mean and peak amplitudes as well as the maximum and minimum slopes of the original spike to be certain that all action potentials were preserved. The PVN or rVLM cells were subjected to spike height discrimination prior to coherence analysis. Autospectra of PVN or rVLM discharge and arterial blood pressure were generated with the FFT. Coherence was generated with seven overlapping windows at 50%, each with a length of 12.8 s, consisting of 256 bins and bin widths of 50 ms. The auto-spectral analysis was adopted from Shin *et al*.^58^, using contiguous segments of 256 beats with 50% overlap between contiguous segments. The frequency resolution was 1/12 s or 0.08 Hz. The coherence function (normalized cross-spectrum) provided a measure of the strength of linear correlation of PVN or rVLM neuronal activity and blood pressure at each frequency. Coherence values of ≥0.5 were chosen to reflect a statistically significant relationship between PVN or rVLM spikes and arterial blood pressure^8^,^46^,^58^,^59^,^60^.

Splanchnic evoked activity was used to quantify neuronal activity during sympathoexcitatory cardiovascular reflex responses. Peristimulus time histograms of neurons were measured with repeated splanchnic afferent stimulation for 15-s at 2 Hz. Evoked activity of neurons was determined as the difference in prestimulus activity (spikes/30stim, duration of 100 ms) and activity at peak response (spikes/30stim, for 100 ms)^26^,^46^.

### Anatomical studies

#### Microinjection of a retrograde tracer into rVLM

To anatomically examine for direct projections between the PVN and rVLM that might be relevant to opioids, using stereotaxic positioning to guide placement of the tip of the injection pipette in the medulla in the region of the rVLM, we microinjected a retrograde tracer in five rats (350–500 g). Previous studies have shown that rats and cats display similar cardiovascular and autonomic responses to EA and that their central neural anatomy and circuits that process somatic input as well as the participating neurotransmitter systems involved during EA are virtually identical^5^,^8^,^44^,^45^,^46^. As we described previously^5^,^8^, a mixture of ketamine-xylazine (80:12 mg/ml, Sigma) was used to induce (0.3–0.4 ml im) and maintain (0.1–0.2 ml im) anesthesia. Body temperature was monitored with a rectal probe and was maintained at 37 °C. Heart rate and oxygen saturation were monitored using a pulse oximeter (Nonin Medical, Plymouth, MN). The rat was placed in a stereotaxic apparatus (David Kopf Instruments). A 1-in. incision was made to expose the skull. A burr hole (4 mm diameter) was made in the occipital bone, according to the following coordinates: 12.0–12.5 mm caudal from the bregma, 2.0–2.5 mm from the midline, and 8.5 mm deep from the dural surface. Fifty nanoliters of a retrogradely transported tracer, rhodamine-labeled fluorescent microspheres in suspension (0.04 μm, Molecular Probes, Eugene, OR), were injected into the rVLM through a glass micropipette. The wound was sutured shut. The microspheres were transported during the 10- to 12-day recovery and maintenance period.

Terminal procedures occurred 10–12 days after administration of the retrograde tracer. Rats were anesthetized deeply with a large dose of the ketamine-xylazine (0.5–0.7 ml im). Transcardial perfusion was performed using 500 ml of 0.9% saline solution followed by 500 ml of 4% paraformaldehyde. The hypothalamus and medulla oblongata were harvested and sliced into coronal sections with a cryostat microtome (Leica CM1850 Heidelberger Strasse, Nussloch, Germany). The hypothalamus was sectioned (30 μm), and neurons labeled with retrograde microsphere tracer and stained for β-endorphin or μ-opioid receptors. Sections of the medulla oblongata (50 μm) were scanned to identify the sites of microinjection of the microsphere tracer.

#### Labeling for β-endorphins and μ-opioid receptors

After washing for 30 min (10 min × 3 times) in phosphate-buffered saline containing 0.3% Triton X-100 (PBST; pH = 7.4), hypothalamic sections were placed for 1 h in 1% normal donkey serum (Jackson Immunoresearch Laboratories, West Grove, PA). The sections were incubated with a primary rabbit polyclonal anti-β-endorphin antibody (1:400 dilutions) or anti-μ-opioid receptor antibody (1:500 dilutions; both antibodies purchased from Chemicon International, Temecula, CA) at 4 °C for 48 h. The tissues subsequently were rinsed three times (10 min for each rinse) in PBST and incubated with fluorescein-conjugated donkey anti-rabbit antibody (1:100; Jackson Immunoresearch Laboratories) for 24 h at 4 °C. Each section was mounted on a slide and was air dried. The slides were coverslipped using mounting medium (Vector Laboratories, Burlingame, CA). Immunohistochemical control studies were performed by omission of the primary or secondary antibodies and by preabsorption with excess (10 μg/ml) β-endorphin or μ-opioid receptor protein. No labeling was detected under the latter conditions. Brain sections were scanned and examined with a standard fluorescent microscope (Nikon, E400, Melville, NY). Two epifluorescence filters (B-2A or G-2A) equipped in a fluorescent microscope were used to identify single stains appearing as green (fluorescein) or red (rhodamine) in brain sections. Sections containing the PVN were identified according to their best matched standard stereotaxic plane, as shown in Paxinos and Watson's atlas for the rat^31^.

After examination with a fluorescent microscope, selected sections were further evaluated with a laser scanning confocal microscope (Zeiss LSM 510, Meta System, Thornwood, NY) to confirm relationship between two labels. This apparatus was equipped with Argon and HeNe lasers and allowed operation of multiple channels. Lasers of 488- and 543-nm wavelengths were used to excite fluorescein (green) and rhodamine (red), respectively. Digital fluorescent images were captured and analyzed with software (Zeiss LSM) provided with the confocal microscope. Each confocal section analyzed was limited to 0.5 μm thickness in the Z-plane. Images containing two colors in the same plane were merged to reveal the relationship between two labels (see Fig. 6). Single- and double-labeled neurons were evaluated.

## Additional Information

**How to cite this article**: Tjen-A-Looi, S. C. *et al*. Paraventricular Nucleus Modulates Excitatory Cardiovascular Reflexes during Electroacupuncture. *Sci. Rep.* **6**, 25910; doi: 10.1038/srep25910 (2016).
